# Chronic Fatigue in Cancer Survivorship: Psychiatry Versus Oncology or Psychiatry with Oncology?

**DOI:** 10.1007/s11912-025-01697-9

**Published:** 2025-06-11

**Authors:** Vasilios Kafetzopoulos, Maria Pittaka, Georgios Ioannidis, Ivi Moniem

**Affiliations:** 1 Department of Psychiatry, Medical School, University of Cyprus, Nicosia, Cyprus; 2 Beth Israel Deaconess Medical Center, Harvard Medical School, Boston, MA USA; 3 Oncology Department, Nicosia General Hospital, State Health Services Organization, Nicosia, Cyprus

**Keywords:** Survivorship, Cancer related fatigue, Myalgic encephalomyelitis/Chronic fatigue syndrome (ME/CFS), Multidisciplinary, Guidelines, Psychiatry

## Abstract

**Purpose of Review:**

Cancer-related fatigue (CRF) is a prevalent and debilitating symptom among breast cancer survivors​ with a significant adverse impact on quality of life​. This comprehensive review synthesizes the current understanding of CRF’s complex pathophysiology, including the interplay of inflammatory, neuroendocrine, and psychosocial mechanisms​, and evaluates diverse intervention strategies.

**Recent Findings:**

Non-pharmacologic approaches (e.g., structured exercise, cognitive-behavioural therapy, mindfulness) have the strongest evidence for alleviating CRF and are emphasized as first-line treatments in oncology guidelines (e.g., ASCO, NCCN, ESMO). In contrast, pharmacologic options such as psychostimulants or bupropion show only modest benefits​, with mixed efficacy and notable side effects​, underscoring their limited role. Comparing oncology-focused guidelines with those for myalgic encephalomyelitis/chronic fatigue syndrome (ME/CFS) which are psychiatry-focused highlight key differences in management approaches and the need for a unified, multidisciplinary framework across specialties.

**Summary:**

Modern multidisciplinary, individualized survivorship care, integrating oncologic, psychosocial, and rehabilitative strategies call for adoption of updated, integrated clinical guidelines to optimally address CRF​. By consolidating evidence and expert recommendations, this review aims to inform and enhance the clinical management of CRF and improve survivorship outcomes for breast cancer survivors.

## Key Points

• **Multifactorial Nature of CRF:** In breast cancer survivors, chronic fatigue is multifactorial, driven by an interplay of persistent biological changes (e.g., cytokine-mediated inflammation, HPA axis and neurotransmitter dysregulation, altered muscle metabolism) and psychosocial factors​.

• **Effective non-pharmacologic Interventions:** Robust evidence supports non-pharmacologic approaches for fatigue management: structured exercise programs and cognitive-behavioural therapy (CBT) significantly alleviate cancer-related fatigue, improving physical function and quality of life in survivors​.

• **Selective Role of Medications:** Pharmacologic interventions play a narrow role in CRF; psychostimulants (e.g., methylphenidate) or bupropion may offer modest fatigue relief in select cases, but inconsistent efficacy and side effects preclude routine use​.

• **Contrasting Guidelines and Integration:** Oncology guidelines prioritize active rehabilitation (exercise and psychosocial interventions) for fatigue​, whereas psychiatric/ME-CFS guidelines emphasize pacing and avoiding overexertion​; this divergence highlights opportunities for interdisciplinary learning and potential convergence of care approaches. Importantly, clinically significant overlap between those entities may occur and be underestimated.

## Introduction

Cancer-related fatigue (CRF) in breast cancer survivors is a distressing and persistent sense of physical, emotional, and cognitive exhaustion related to cancer or its treatment, disproportionate to activity level and not relieved by rest [1]. It is one of the most prevalent and debilitating long-term symptoms among cancer survivors. For example, approximately 25–40% of disease-free breast cancer survivors report severe fatigue years after completing treatment​, a rate significantly higher than among age-matched individuals without cancer [2–4]. This enduring fatigue can substantially impair quality of life and daily functioning, limiting survivors’ ability to resume normal activities and work [5]. Unlike acute fatigue, CRF does not resolve with sleep and can persist for months or years post-therapy [6]. It often coexists with other sequelae of cancer treatment (such as sleep disturbance or mood changes), compounding its impact on well-being. Given its prevalence and impact, addressing chronic fatigue has become a critical component of survivorship care [4].

Cancer-related fatigue (CRF) in breast cancer survivors is a distressing and persistent sense of physical, emotional, and cognitive exhaustion related to cancer or its treatment, disproportionate to activity level and not relieved by rest [1]. It is one of the most prevalent and debilitating long-term symptoms among cancer survivors. For example, approximately 25–40% of disease-free breast cancer survivors report severe fatigue years after completing treatment​, a rate significantly higher than among age-matched individuals without cancer [2–4]. This enduring fatigue can substantially impair quality of life and daily functioning, limiting survivors’ ability to resume normal activities and work [5]. Unlike acute fatigue, CRF does not resolve with sleep and can persist for months or years post-therapy [6]. It often coexists with other sequelae of cancer treatment (such as sleep disturbance or mood changes), compounding its impact on well-being. Given its prevalence and impact, addressing chronic fatigue has become a critical component of survivorship care [4].

Emerging recognition of CRF has led to its inclusion in survivorship guidelines and research agendas. Major oncology organizations now recommend routine screening for fatigue at follow-up visits and prompt intervention when fatigue is moderate or severe [7]. Unlike many acute treatment toxicities, CRF may remain or even arise well into the post-treatment period, making long-term management essential. The remainder of this review will discuss the pathophysiology and clinical features of CRF, evidence-based treatment approaches, current guideline recommendations from oncology and psychiatry perspectives, and future directions in research and care. Throughout, the focus is on chronic fatigue in cancer survivors and breast cancer survivors specifically, excluding issues specific to active treatment or other symptoms. Our aim is to provide a comprehensive, up-to-date analysis of chronic fatigue in cancer survivorship and to offer practical insights for improving outcomes in this challenging population.

This review will discuss the pathophysiology and clinical features of CRF, evidence-based treatment approaches, current guideline recommendations from oncology and psychiatry perspectives, and future directions in research and care. Throughout, the focus is on chronic fatigue in cancer survivors, excluding issues specific to active treatment or other symptoms.

Our aim is to provide a comprehensive, up-to-date analysis of chronic fatigue in cancer survivorship and to offer practical insights for improving outcomes in this challenging population.

### Pathophysiology and Clinical Features

#### Biological Mechanisms

The etiology of CRF is multifactorial, involving complex interactions between biological and non -biological psychosocial processes. A leading hypothesis implicates persistent immune activation and inflammation triggered by cancer and its treatment [8]. Survivors with CRF often exhibit elevated levels of pro-inflammatory cytokines (e.g. IL-6, TNF-α) and C-reactive protein, supporting an “inflammatory signature” in fatigue [8, 9]. These cytokines can signal the central nervous system to induce fatigue and sickness behavior, a pathway analogous to mechanisms seen in chronic inflammatory and infectious conditions [10].

**Fig. 1:**
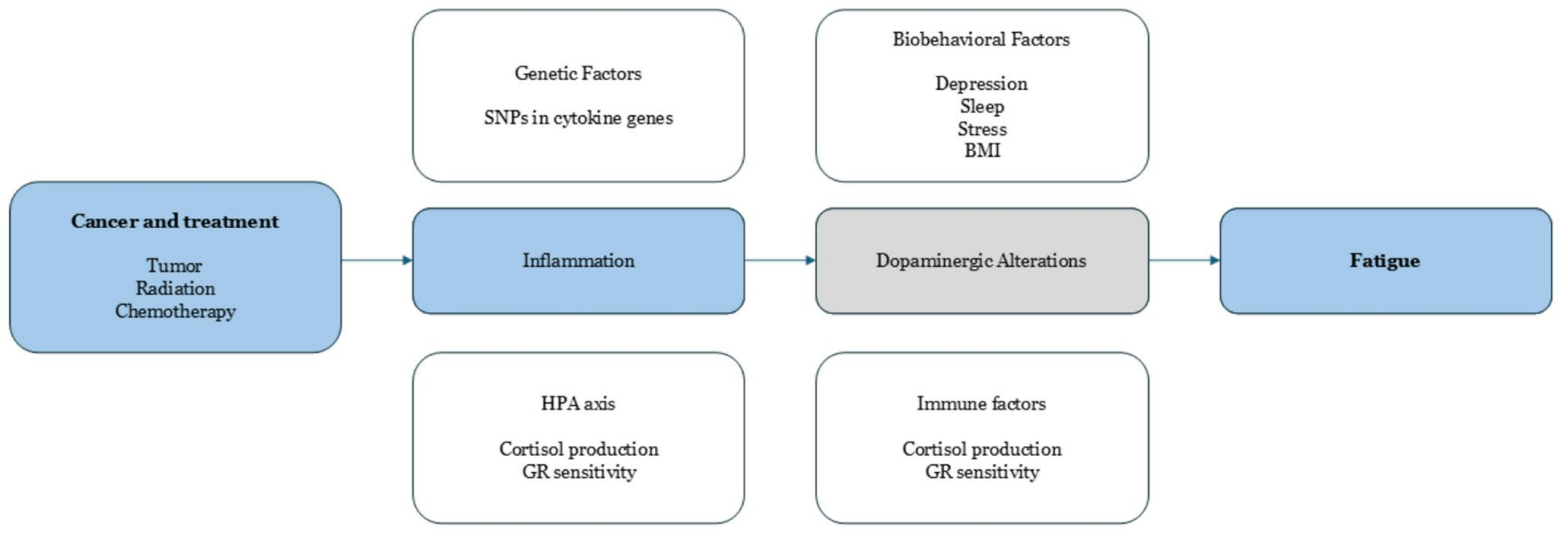
Conceptual model linking cancer and cancer treatments to inflammation and fatigue, with potential mediating pathways and host factors (adapted with permission from Bower et al., 2011). Pro-inflammatory cytokine activity triggered by the tumor or therapy can lead to central fatigue via neurotransmitter changes (e.g. dopaminergic alterations). Host factors (non colored) – including genetic variants, HPA axis and immune system changes, and biobehavioral factors (such as depression, sleep disturbance, stress, and high body mass index) – may influence the onset and persistence of inflammation and fatigue

Literature suggests a conceptual model in which cancer and its treatments initiate an inflammatory cascade that leads to neurotransmitter alterations (notably in dopaminergic pathways) and fatigue​ [11]. More specifically, host factors such as genetic predispositions (e.g. cytokine gene polymorphisms), hypothalamic-pituitary-adrenal (HPA) axis dysfunction, and comorbid medical conditions can modulate this inflammation-fatigue pathway​ [12]. For instance, blunted HPA axis responsiveness or mitochondrial dysfunction may contribute to fatigue by impairing stress tolerance and cellular energy production [13]. Additionally, cancer treatments can produce direct physiologic deficits – for example, chemotherapy-induced anaemia or thyroid dysfunction – which exacerbate fatigue. Reversible medical contributors such as anaemia and endocrine disorders should be evaluated in any survivor reporting significant fatigue​ [14]. Figure 1 illustrates this model.

Beyond inflammation, other biological mechanisms have been proposed. Dysregulation of the HPA axis (e.g. abnormal cortisol rhythms or glucocorticoid receptor sensitivity) has been observed in fatigued survivors [15]. Mitochondrial DNA damage and impaired ATP synthesis in skeletal muscle have also been reported, suggesting a component of cellular energy depletion in CRF [16, 17]. Autonomous dysregulation can also account for some of the perceived or objective disturbances seen by those patients [18]. Additionally, premature ovarian failure or androgen deprivation (from cancer therapies) can produce hormonal changes associated with fatigue – for example, abrupt menopause in women or testosterone deficiency in men may precipitate lethargy and muscle weakness. These diverse biological factors are not mutually exclusive; they likely interact in contributing to an individual patient’s fatigue.

#### Psychosocial Factors and Symptom Overlap

Psychological and behavioural factors are equally important in CRF. Fatigue in survivors is strongly correlated with mood disturbances (especially depression and anxiety), poor sleep, and cognitive impairment [19]. Survivors with clinically significant fatigue often have co-occurring insomnia or disrupted sleep architecture, which can perpetuate daytime fatigue in a vicious cycle. Likewise, there is substantial overlap between fatigue and depression symptoms (e.g. low energy, diminished motivation) [20]. However, it is noted that many fatigued survivors do *not* meet criteria for major depression – indicating that CRF is not simply a manifestation of depression, though the two can coexist. When depression is present, it can worsen fatigue intensity, and treating the mood disorder may partially relieve fatigue [21].

Conversely, standard antidepressants (e.g. SSRIs) do not consistently improve fatigue in non-depressed patients and in some cases can cause fatigue as a side effect [22, 23]. This underscores the need to distinguish CRF from depression and to address each appropriately.

Cognitive-behavioural factors also play a role: survivors may reduce activity in response to fatigue, leading to deconditioning, which then further exacerbates fatigue. Lack of exercise, prolonged rest, and social withdrawal can all reinforce fatigue over time. Indeed, feelings of helplessness or lack of actionable solutions about fatigue have been associated with worse outcomes, whereas active coping strategies such as pacing and scheduling meaningful activity when energy is highest are linked to lower fatigue [24]. Table 1 summarizes key factors in pathogenesis of CRF.

**Table 1:** Key contributing factors in the pathogenesis of Cancer-Related fatigue (CRF). CRF is typically **multifactorial**, and individual patients May have a unique mix of contributing factors. Comprehensive assessment is recommended to tailor interventions [33]

Factor	Description and Role in CRF	Key evidence (References)
Inflammatory cytokines	Elevated IL-1, IL-6, TNF-α promote fatigue via CNS effects (sickness behavior).	[25, 9]
HPA axis dysfunction	Abnormal cortisol rhythms and stress response contribute to fatigue.	[15]
Neurotransmitter changes	Increased central serotonin, disrupted dopamine/norepinephrine linked to fatigue.	[26, 24]
Neural inflammation	Activation of microglia, reduced neurogenesis may sustain fatigue signals in brain.	[25, 18]
Muscle loss & dysfunction	Cancer/cachexia and chemo cause muscle wasting; mitochondrial damage reduces ATP (energy).	[16, 17]
Anaemia (low hemoglobin)	Reduces oxygen delivery leading to weakness; common during treatment.	[27]
Sleep disturbance	Insomnia or fragmented sleep intensifies fatigue due to non-restorative rest.	[28, 19]
Psychological distress	Depression and anxiety amplify fatigue perception and vice versa (bi-directional).	[29, 30]
Deconditioning	Reduced activity -> loss of fitness -> more fatigue (vicious cycle of inactivity).	[31, 32]

### Clinical Features

Patients describe CRF as profound tiredness that is disproportionate to effort and not relieved by sleep. They often experience concentration difficulties (“chemo brain”), memory lapses, and slowed thinking alongside the physical exhaustion [34]. Many survivors note that mental fatigue – an inability to focus or remain alert – is as troublesome as the loss of physical stamina. Unlike normal fatigue, which is predictable and improves with rest, CRF can persist day after day regardless of sleep and can fluctuate unpredictably. CRF typically has an insidious onset, either gradually during treatment or emerging in the months after treatment completion. Clinically, it is important to evaluate for other causes (recurrent disease, thyroid dysfunction, cardiopulmonary issues, etc.) when survivors report new or worsening fatigue. Most cases of CRF are diagnosed by exclusion of such medical causes combined with patient-reported outcomes [22].

Accurate **assessment of CRF** is essential to guide management. CRF is subjective, so **patient self-report measurement is the gold standard** [35]. Common approaches include asking patients to rate fatigue intensity (0–10 scale) and using validated questionnaires. Examples of multi-dimensional fatigue instruments are the **Brief Fatigue Inventory (BFI)**, **Multidimensional Fatigue Inventory (MFI)**, and **Functional Assessment of Chronic Illness Therapy-Fatigue (FACIT-F)** and EORTC QLQ-FA12 in conjunction with the EORTC QLQ-C30 questionnaire [36–38]. These tools assess fatigue severity, duration, and impact on daily function.

Clinical guidelines recommend **screening at regular intervals**, such as at diagnosis, throughout treatment, and during survivorship regular consultations. In BFI, a **score ≥ 4/10** often signals clinically significant fatigue warranting intervention, with 0 signifying no fatigue, 1–3 mild fatigue, 4–6 moderate fatigue and 7–10 severe fatigue [37] For individuals > 12 years old, on a 0 to 10 numeric rating scale (0 = no fatigue and 10 = worst fatigue imaginable), mild fatigue is indicated as a score of 1 to 3, moderate fatigue as 4 to 6, and severe fatigue as 7 to 10. The evaluation of fatigue in children aged 7 to 12 may be simplified to a scale of 1 to 5 and modified even further in young children (aged 5–6 years) who may be asked more simply if they are “tired” or “not tired.” [39].

The multidimensional pathogenesis means that chronic fatigue must be approached from multiple angles. The multifactorial nature of CRF also explains why there is no simple cure – effective management usually requires a *comprehensive*,* interdisciplinary strategy* addressing both the body and mind. Importantly, while CRF can be prolonged, it is a manageable condition. Even if complete resolution is not immediate, meaningful improvements in energy levels and functioning are achievable with proper interventions, as discussed below. Table 2 highlights select risk factors associated with CRF.

**Table 2:** Selected risk factors associated with increased Cancer-Related fatigue. The risk factors include demographical, personal, cancer and treatment variables, both modifiable and unmodifiable

Risk Factor	Association with Fatigue	Supporting Evidence
Younger age	Younger adult patients often report higher fatigue than older patients, potentially due to intensive treatments and lifestyle disruptions.	[6]
Female gender	Some studies show women experience more fatigue (may reflect breast cancer cases or reporting differences).	[29]
Lack of social support	Patients without a partner or strong support network have higher risk of severe fatigue post-treatment.	[40]
Advanced cancer stage	Metastatic or advanced disease correlates with higher fatigue prevalence and severity.	[41]
Cancer type	Cancers with high inflammatory burden (e.g., leukemia, lymphoma) or common anaemia (e.g., GI cancers) can cause more fatigue.	[42]
Chemotherapy (multi-agent)	Combination regimens and certain drugs (e.g., doxorubicin, cyclophosphamide, platinum, taxanes) increase fatigue more than single agents or less toxic drugs.	[43, 44]
Radiation therapy	Fatigue is common during radiation (especially extensive fields or concurrent chemoradiation).	[45, 46]
Hormonal or targeted therapy	Endocrine treatments and cytokine-based immunotherapies can induce fatigue (via metabolic or immune pathways).	[29, 140]
Multiple comorbidities	Co-existing conditions (heart, lung, kidney disease, etc.) compound fatigue and limit compensatory capacity.	[36]
Psychological distress	Patients with pre-existing or concurrent depression/anxiety are more prone to severe fatigue.	[30, 29]
Sedating medications	Use of opioids, benzodiazepines, or other CNS depressants can worsen fatigue levels.	[47]

### Treatment Options for Chronic Fatigue

#### Non-Pharmacological Interventions (First-Line)

Management of cancer-related fatigue in cancer begins with conservative approaches, especially when cancer survivors are getting progressively younger as a demographic group [48]. All major guidelines recommend non-pharmacologic strategies as first-line therapy for CRF in survivors [33, 49]. These include **exercise** (both aerobic and resistance training), which has the strongest evidence base, as well as behavioural and psychosocial interventions such as **cognitive-behavioural therapy (CBT)**, **mindfulness-based stress reduction**, **yoga**, and **psychoeducation**. A robust body of randomized trials and meta-analyses has shown that supervised exercise programs can significantly improve fatigue in cancer survivors, increasing strength and endurance and breaking the cycle of deconditioning​ [50, 51]. Psychosocial interventions like CBT specifically tailored to fatigue have also demonstrated efficacy by addressing unhelpful fatigue-related thoughts, improving coping skills, and treating co-occurring depression or sleep problems. For example, the American Society of Clinical Oncology (ASCO) and Society for Integrative Oncology’s updated 2024 guidelines strongly endorse exercise, CBT, and mindfulness-based therapies as effective interventions for fatigue in both active cancer treatment and post-treatment survivorship​ [35]. Mind-body practices such as tai chi and qigong have shown benefits during treatment, and yoga and acupressure have evidence for reducing fatigue in the post-treatment setting​.

In contrast, **rest alone is not a cure** for CRF – in fact, excessive rest may worsen conditioning and fatigue. Thus, survivors are encouraged to engage in a balanced program of physical activity (tailored to their abilities) combined with energy conservation techniques (pacing of activities and planned rest), rather than prolonged inactivity. Sleep hygiene and stress management are also important components, since improving sleep quality and reducing anxiety can alleviate fatigue. A meta-analysis by [50] compared different classes of fatigue interventions and found that exercise and psychological interventions produced improvements in fatigue that were at least as large as (and in some cases greater than) those produced by pharmacologic treatments [23]. Given the favourable risk-benefit profile of behavioural interventions, these are the preferred initial approach. In practice, a multimodal plan – incorporating exercise, psychosocial support (e.g. support groups or counselling), and symptom management (for pain, insomnia, etc.) – is recommended for most breast cancer survivors with persistent fatigue. Only when fatigue remains moderate-to-severe despite these measures, or when patients are unable to fully engage in non-pharmacological strategies, should pharmacologic options be considered as adjuncts [52]. Before initiating any medication for fatigue, clinicians should also evaluate and treat contributing medical factors (thyroid levels, anaemia, uncontrolled pain, etc.), since correcting those can sometimes markedly improve fatigue without the need for drug therapy [53]. With those caveats, several pharmacological approaches have been explored for CRF.

#### Psychostimulants and Wakefulness-Promoting Agents

Psychostimulant medications have been among the most studied pharmacologic treatments for cancer-related fatigue. The rationale is that stimulants enhance dopaminergic and adrenergic signalling, potentially counteracting the lethargy and poor concentration associated with CRF [54]. The two agents tested most extensively are **methylphenidate** (a traditional CNS stimulant used for ADHD) and **modafinil** (a wakefulness-promoting drug used for narcolepsy). Early studies, including small randomized trials and case series in cancer patients, suggested these stimulants might reduce fatigue and improve cognitive focus [55]. For example, an initial double-blind trial in advanced cancer patients reported that methylphenidate led to significant short-term fatigue relief compared to placebo [56]. Similarly, an open-label pilot study in breast cancer patients on chemotherapy found modafinil subjectively improved energy and alertness [57]. These promising early results generated enthusiasm; however, larger rigorous trials yielded more mixed findings.

A *landmark* phase III trial (URCC CCOP study) tested modafinil in 631 patients receiving chemotherapy (including many breast cancer patients), and found that modafinil significantly reduced fatigue only in the subset of patients who had **severe** baseline fatigue – those with moderate fatigue did not derive a clear benefit​ [58]. In other words, modafinil helped the worst-fatigued patients but was no better than placebo for milder fatigue. Another placebo-controlled trial of modafinil in patients with lung cancer (who often experience higher symptom burden compared to other cancer types) showed **no significant improvement** in fatigue levels overall [59] tempering early optimism.

Methylphenidate has likewise shown **inconsistent** results. Some randomized trials in advanced cancer or palliative care settings have reported that methylphenidate yields modest short-term fatigue improvement – for instance, a study of patient-controlled methylphenidate in advanced cancer observed reduced fatigue in the methylphenidate arm [60]. A 2018 meta-analysis by Tomlinson et al. examined pharmacologic interventions for fatigue across cancer populations (including transplant patients) and found that psychostimulants (primarily methylphenidate) were associated with a small but statistically significant reduction in fatigue compared to placebo. The pooled effect size was around 0.30, indicating a small benefit [52]. However, there was substantial heterogeneity between studies. More recently, a comprehensive systematic review and meta-analysis focusing specifically on cancer survivors (Sun et al., 2021) concluded that psychostimulants like modafinil and methylphenidate do produce a **measurable improvement** in fatigue scores relative to placebo [61]. Importantly though, the **magnitude** of improvement was **clinically small** – on the order of a 2–3 point change on 0–10 fatigue scales, which is below the threshold usually considered a minimally important difference. In practical terms, this means that while some patients feel a bit better on stimulants, the average benefit may be modest and not everyone responds. These findings suggest stimulants are not a panacea, but they *can* help a subset of survivors, particularly those with severe fatigue who have not responded to other measures​. In clinical experience, certain patients describe a notable boost in energy and ability to function with these medications, whereas others have little effect or cannot tolerate the side effects.

Side effects of psychostimulants are indeed an important consideration. Within others, they can cause insomnia, jitteriness, anxiety, appetite loss, headache, or heart palpitations, among other effects [62]. Typically, low doses are used (e.g. methylphenidate 5–10 mg in the morning, titrating up as needed) and patients are monitored closely. If no improvement in fatigue is seen after a trial period (say 4–6 weeks), the drug should be discontinued [63]. Current practice guidelines reflect the *reserved* role of stimulants. Earlier oncology guidelines (e.g. National Comprehensive Cancer Network, NCCN) acknowledged that methylphenidate or modafinil may be **considered** in patients with persistent, severe fatigue after addressing other factors. For example, NCCN guidelines have noted that a psychostimulant can be offered, preferably for a time-limited trial, in refractory cases – particularly in advanced cancer or end-of-life settings where immediate fatigue relief is a priority [54]. The European Society for Medical Oncology (ESMO), however, has been more cautious, not recommending routine use of stimulants due to insufficient evidence [33]. Notably, the latest ASCO–SIO 2024 survivorship fatigue guideline explicitly states that clinicians **should not** prescribe **psychostimulants**,** wakefulness agents (like modafinil)**,** or antidepressants** to manage CRF in post-treatment survivors, given the weak overall efficacy data [64]. This represents a strong stance against routine stimulant use in this context. In practice, some oncologists and psycho-oncology specialists still employ stimulants on a case-by-case basis – especially if a patient is profoundly fatigued and other interventions have failed. In such cases, a careful informed discussion is needed. Clinicians typically rule out medical causes (e.g. recurrence, severe anaemia, etc.), then initiate a low-dose stimulant with close follow-up [65]. If meaningful benefit is observed (e.g. improved daily functioning) and side effects are tolerable, the medication might be continued, but if not, it is discontinued. The overall takeaway is that psychostimulants *can* offer symptomatic relief for CRF in some patients, but expectations should be modest, and they are not universally effective. Non-pharmacologic therapies should remain the cornerstone, with drugs like methylphenidate or modafinil reserved for refractory fatigue after other measures.

#### Antidepressants and Other Neuromodulators

Another pharmacologic approach has been repurposing antidepressant medications to manage fatigue. This stems from the overlap between fatigue and depressive symptoms, and the hypothesis that enhancing certain neurotransmitters (dopamine, norepinephrine) might increase energy and motivation. However, traditional antidepressants that act primarily on serotonin – such as SSRIs – have generally **not** shown benefit for CRF unless the patient has a diagnosable concurrent depression​ [50]. In non-depressed cancer survivors, SSRIs (e.g. paroxetine, sertraline) have largely failed to improve fatigue and in some cases can *worsen* fatigue as a side effect (due to sedative properties) [66]. Thus, there is little support for routine use of SSRIs purely for fatigue. On the other hand, **bupropion** has emerged as a promising agent. Bupropion is an atypical antidepressant that inhibits dopamine and norepinephrine reuptake, effectively a “mild stimulant” in its pharmacologic profile (it is structurally similar to amphetamine), thus a logical candidate for treating fatigue. Several studies have tested bupropion in cancer-related fatigue with encouraging results. A small, randomized placebo-controlled trial in 2004 (Cullum et al.) first reported that sustained-release bupropion significantly reduced fatigue in a mixed sample of cancer patients [67]. More recently, two randomized trials focused on fatigued breast cancer survivors: one by Ashraf et al. (2018) and one by *Salehifar et al.* (2020). Both trials found that a moderate dose of bupropion SR (typically 150 mg daily) led to greater improvements in fatigue scores over ~ 6 to 8 weeks compared to placebo​ [68, 69]. In these studies, patients on bupropion reported increased energy and better ability to concentrate relative to the control group. While these trials were relatively small (each < 100 participants), their positive findings align with clinical anecdotes that some patients feel “less drained” on bupropion. Bupropion is generally well-tolerated; its side effect profile (activating, with low sexual side effects and no weight gain) can counteract some issues survivors face. It can, however, cause insomnia or anxiety in susceptible individuals, so timing (morning dosing) and monitoring are needed. Psychotropics with cytochrome P450 interactions (i.e., fluoxetine, paroxetine, sertraline, bupropion, fluvoxamine, nefazodone duloxetine, clomipramine) should be used with caution in survivors taking tamoxifen. Pure SSRIs, and in particular paroxetine, block conversion of tamoxifen to active metabolites through CYP2D6 and should be used with caution for patients on tamoxifen [70].

Recognizing the need for more definitive evidence, a larger U.S. trial known as the “ENERGIZE” study has been designed to rigorously test bupropion for CRF in cancer survivors [71]. This phase III randomized trial (sponsored by the National Cancer Institute) is enrolling fatigued cancer survivors to receive bupropion or placebo, with results expected to clarify its efficacy on a larger scale. If positive, bupropion could become a more standard pharmacologic option for fatigue. Given its dual benefits on mood and energy, bupropion may be particularly useful for patients who have *both* mild depression and fatigue—a common scenario in survivorship​. It effectively addresses two symptoms at once in such cases. Even in the absence of major depression, bupropion might help those who experience fatigue along with brain fog or daytime sleepiness (sometimes sequelae of chemotherapy or long-term endocrine therapy).

Another agent that has been evaluated is **amantadine**, an older dopaminergic drug originally used in Parkinson’s disease and for fatigue in multiple sclerosis. Amantadine has dopamine-releasing and NMDA-antagonist properties that could theoretically combat fatigue. Small trials in cancer patients have yielded mixed outcomes. A 2015 systematic review and meta-analysis (Kronenberg et al.) examined amantadine’s effects on CRF and found inconsistent results across studies, with no clear evidence of a significant benefit [72]. Some patients appeared to improve on amantadine, but others did not, and side effects (such as insomnia and nausea) were noted. At present, amantadine is not commonly used for CRF outside of experimental contexts. Ongoing comparative research may shed light on its relative efficacy. Notably, an international multi-arm trial called “5-EPIFAT” is underway, comparing methylphenidate, modafinil, bupropion, and amantadine (versus placebo) in patients with cancer-related fatigue. This randomized trial will directly evaluate and rank these pharmacologic options in parallel, which should help determine if one agent outperforms others or if all have similarly limited effects. Early-phase results or protocol publications [73] indicate that this study is actively enrolling patients and will provide valuable head-to-head data​.

In practice, when choosing a pharmacological therapy for a survivor with chronic fatigue, clinicians tailor the choice to the patient’s symptom profile and comorbidities. For instance, if a patient’s fatigue is accompanied by depressed mood or apathy, an activating antidepressant like **bupropion** might be preferred. If excessive daytime sleepiness or concentration deficit is prominent (but mood is fine), a **modafinil or methylphenidate** trial could be reasonable. If pain is a big component, optimizing analgesics (e.g. **duloxetine** might help both pain and energy in some cases) is important – although duloxetine is primarily for neuropathic pain, a patient with coexistent depression and fatigue might incidentally benefit in mood/energy from it [74].

Importantly, many of these medications can be used in combination with non-pharmacologic strategies. In fact, a combination approach is often ideal: for example, a short course of a stimulant or bupropion might be used to “jump-start” a severely fatigued patient and enable them to engage in an exercise program that they previously could not due to low energy. Such integrative use of pharmacotherapy, to facilitate participation in foundational therapies like exercise, is a pragmatic strategy some clinicians employ.

#### Other Pharmacologic Measures

A few additional pharmacological approaches deserve brief mention. **Corticosteroids** (like dexamethasone or prednisone) have long been used in palliative cancer care to improve appetite, pain, and energy in the short term. Small trials in advanced cancer patients have shown that low-dose dexamethasone can provide temporary improvement in fatigue and quality of life​ [34]. For example, Yennurajalingam et al. (2013) found a two-week course of dexamethasone modestly reduced fatigue in patients with advanced cancer compared to placebo​ [75]. Steroids likely help by suppressing inflammatory cytokines and perhaps stimulating mood and appetite. However, due to their numerous side effects (insomnia, muscle atrophy, hyperglycaemia, immunosuppression, etc.), steroids are **not a sustainable long-term solution** for cancer survivors with chronic fatigue​ [76]. They are generally reserved for those in end-of-life or metastatic settings where a short burst can improve function during a critical period. ASCO’s 2024 guideline update notes that corticosteroids may be offered for fatigue management *only* in patients at the end of life (when long-term risks are less relevant) [64].

Another area of interest has been **anti-inflammatory therapies**. Given the link between inflammation and CRF, some studies have explored whether anti-inflammatory drugs or cytokine inhibitors could alleviate fatigue. Small trials of NSAIDs or Cox-2 inhibitors have not shown clear benefits for CRF specifically [77]. More targeted approaches, like blocking IL-1 or TNF-alpha, are still experimental. One pilot trial tested an etanercept (TNF blocker) in cancer patients with fatigue, but results were limited to patients actively receiving chemotherapy [78]. Ongoing research is examining if agents like **exercise mimetics** or metabolic modulators (for example, a trial of the metabolic drug DCA – dichloroacetate – was investigated in a mouse model and showed reduced fatigue behaviour​ [79]) could play a role in the future, though none are clinically available yet for fatigue. Nutritional supplements have also been studied – notably **L-carnitine** (an amino acid involved in mitochondrial energy production) was hypothesized to help, but a large placebo-controlled trial found no improvement in fatigue with L-carnitine supplementation [80]. **Vitamin D** correction is important for bone health, but high-dose vitamin D did not show a fatigue benefit beyond treating deficiency [81]. **Herbal therapies** like American ginseng have shown some efficacy in trials (a Mayo Clinic-led RCT found 2000 mg daily of Wisconsin ginseng improved fatigue over placebo) [82], and a meta-analysis suggested ginseng can yield modest fatigue reduction [83]. However, results are inconsistent (another trial of Asian ginseng was negative [84]), and herbal supplements vary in quality; thus, they are not part of mainstream guidelines.

In summary, **no medication is currently approved specifically for CRF**, and pharmacologic treatments generally provide only partial relief. Overall effect sizes tend to be small [61, 85]. Nonetheless, carefully selected medications – **psychostimulants**,** wakefulness agents**,** and certain antidepressants** – can be valuable tools in a multidisciplinary fatigue management plan, especially for patients who do not adequately respond to exercise or psychotherapy alone. The key is to individualize treatment and set realistic expectations. Often, the goal is a **meaningful but not complete** improvement in fatigue (for instance, reducing fatigue from “severe” to “moderate” so that the patient can resume some activities). Medications may be started one at a time, with clear goals and defined trial periods. If no benefit is seen, they should be stopped to avoid polypharmacy and associated potential side effects. If benefit is observed, the lowest effective dose should be used, and regular re-assessment should be performed. Combining approaches is commonly needed – for example, optimizing pain control and sleep, treating depression, encouraging daily walks, and possibly adding a stimulant – all together may yield a cumulative improvement that none of those alone could achieve. Managing CRF often requires this kind of **comprehensive strategy**, reflecting the multifactorial nature of the condition. Indeed, experts emphasize that pharmacotherapy should be just **one component** of a broader fatigue management plan that also addresses exercise, psychosocial support, sleep, nutrition, and comorbid symptoms​ [60].

#### Integrative and Multimodal Approaches

Because CRF has multiple causes, the most successful management plans are typically multimodal. In practice, this means combining physical, psychological, and medical interventions in a personalized manner [86]. For example, a survivor with severe fatigue might be enrolled in a tailored exercise program *and* receive a short course of modafinil to help initiate that program. Another survivor with fatigue plus concurrent anxiety might undergo CBT focused on stress management and sleep, while also practicing yoga and using an alerting agent like bupropion in the morning. Integrating therapies can produce synergistic benefits – exercise improves sleep, better sleep improves fatigue, reduced fatigue allows more exercise, and so on. Close follow-up is essential: fatigue levels should be re-assessed periodically (e.g. monthly) and the plan should be adjusted. If a medication was started, it should be tapered off if no clear benefit; if an exercise regimen is too exhausting, it should be modified to avoid discouragement.

Engaging survivors in their care is paramount: educating patients that improving chronic fatigue often requires active participation (like physical therapy, behaviour change) sets proper expectations and encourages a sense of control. Encouragingly, studies indicate that when patients adhere to combined interventions, a significant proportion achieve clinically meaningful reductions in fatigue [87]. Healthcare providers should also be vigilant about medication side effects or new issues that arise – for instance, watching for stimulant overuse or sleep aid dependence – and employ safeguards such as periodic medication reviews. Ultimately, managing CRF is an ongoing process that may require different tactics as the survivor moves farther out from treatment. A multidisciplinary team – involving oncologists, rehabilitation specialists, psychologists, exercise physiologists, and primary care providers – can collaborate to ensure all aspects of fatigue are addressed. This comprehensive approach gives the best chance of substantially improving CRF and helping survivors regain a high quality of life.

### Guidelines: Oncology Vs. Psychiatry Perspectives

Clinical practice guidelines for cancer-related fatigue have been developed by major oncology organizations, while in the psychiatric domain fatigue is addressed within broader contexts (such as depression or somatic symptom disorders). This has led to some differences in emphasis and approach. Below, we compare key recommendations from oncology-focused guidelines (e.g., NCCN, ASCO, ESMO) with perspectives from psychosocial/psychiatric guidelines and classifications.

#### Oncology Guidelines

Oncologists recognize CRF as a distinct and significant survivorship issue. The National Comprehensive Cancer Network (NCCN) and American Society of Clinical Oncology (ASCO) guidelines on cancer-related fatigue [2, 64] stress routine *screening* for fatigue at each follow-up visit. Fatigue intensity can be quickly assessed on a 0–10 scale; a score of 4 or higher (moderate to severe fatigue) triggers a more thorough evaluation. The next step is to *evaluate and treat contributing factors*. Oncology guidelines outline a checklist of reversible factors to address: unmanaged pain, emotional distress, sleep dysfunction, anaemia, thyroid imbalance, nutritional deficits, etc. (NCCN). For example, if a survivor is hypothyroid or anaemic, that should be corrected as it may markedly improve fatigue. If pain or insomnia is present, appropriate pain management or sleep therapy should be instituted, since untreated pain or sleep loss can worsen fatigue. This comprehensive medical assessment is a cornerstone of oncology guidelines – it ensures that CRF is not simply accepted as inevitable when a fixable issue might be driving it. Once contributing factors are managed, the *first-line interventions* for CRF per oncology guidelines are non-pharmacologic: namely **exercise**,** psychosocial interventions**,** and education** [64].

The **ESMO (European Society for Medical Oncology)** Clinical Practice Guidelines concur, stating that individualized exercise programs and psychological support (such as CBT) should be offered to all cancer survivors with fatigue [33]. Patients should be counselled that moderate physical activity is safe and beneficial, and referrals to physiotherapy or cancer rehabilitation services are encouraged to facilitate exercise. Oncology guidelines also recommend teaching energy conservation techniques and optimizing sleep hygiene for all patients. Use of **pharmacotherapy** is generally reserved for specific indications. The NCCN guidelines note that psychostimulants (like methylphenidate) may be considered for short-term use in carefully selected patients with severe fatigue, but they do *not* endorse long-term stimulant use due to the limited efficacy data and potential adverse effects. ASCO’s guideline adaptation similarly advises that there is insufficient evidence to routinely prescribe medications for CRF, apart from treating underlying conditions (like antidepressants for depression, or erythropoietin for chemotherapy-induced anaemia). Essentially, the oncology stance is: *manage medical causes*,* maximize non-pharmacologic therapies*,* and use drugs sparingly.* There is acknowledgment that some patients may benefit from stimulants or other agents, but these should be individualized decisions and ideally within a clinical trial or after other approaches fail. All guidelines emphasize the importance of *multidisciplinary care* – involving oncologists, nurses, mental health professionals, physical therapists, and others in a coordinated fatigue management plan. The table below summarizes key aspects of oncology guideline recommendations (Table 3).

**Table 3:** Oncology guidelines by NCCN, ASCO and ESMO summarized for every key aspect of symptomatology and recommended course of action. Oncology guidelines are increasingly evidence-based as research in CRF grows. For example, the 2020 update of the ESMO guidelines incorporated recent trials by recommending specific aerobic exercise prescriptions and noting that yoga and mindfulness have supportive evidence. These guidelines also highlight that one approach does not fit all – interventions should be tailored to cancer type, treatment history, and patient preferences. Notably, oncology guidelines frame CRF management as an ongoing process throughout survivorship, not a one-time treatment [2, 33, 64]

Aspect	Details
Oncology Guidelines	NCCN, ASCO, ESMO
Recognition of CRF	Defined as a distinct condition requiring attention. Routine screening at each visit (e.g., ask patient to rate fatigue 0–10). Fatigue is considered the “6th vital sign” in survivorship.
Evaluation	Defined as a distinct condition requiring attention. Routine screening at each visit (e.g., ask patient to rate fatigue 0–10). Fatigue is considered the “6th vital sign” in survivorship.
First-line Therapy	Non-pharmacologic interventions for all patients: exercise (tailored to patient ability), behavioural therapy (e.g. CBT for fatigue, counselling), and education on energy conservation and sleep hygiene. Encourage physical activity as tolerated; refer to rehabilitation or supervised exercise programs. Provide psychosocial support (stress management, support groups).
Pharmacologic Use	No routine drug therapy for CRF. Consider psychostimulants (methylphenidate or modafinil) only for severe fatigue unrelieved by other means, after medical causes addressed. If used, start low dose, use time-limited trial with monitoring. Treat co-morbid depression or anxiety per standard guidelines (e.g., antidepressants) – this can indirectly improve fatigue. Erythropoietin-stimulating agents are only for chemotherapy-induced anaemia with Hb < 10 g/dL, not for CRF generally.
Follow-up & Adjustment	Regular re-assessment of fatigue and function. If interventions are not helping after a few weeks or fatigue worsens, re-evaluate for new contributing factors or adjust the plan. Involve palliative care for persistent severe fatigue impacting QOL. Continue long-term physical activity and self-management strategies even after initial improvement, to maintain gains.

### Psychiatric Guidelines for Chronic Fatigue Syndrome (ME/CFS)

Chronic fatigue that is not attributable to cancer or its treatment is most often discussed in the context of **Myalgic Encephalomyelitis/Chronic Fatigue Syndrome (ME/CFS)** – a complex disorder characterized by persistent fatigue lasting at least 6 months with no alternative medical explanation [88]. Notably, ME/CFS is **distinct from cancer-related fatigue (CRF)** in etiology and management, and formal psychiatric guidelines specific to chronic fatigue (ME/CFS) have been limited. Neither the American Psychiatric Association (APA) nor the World Psychiatric Association (WPA) has issued dedicated clinical guidelines for ME/CFS. In practice, psychiatrists and other clinicians rely on multidisciplinary guidelines and consensus statements developed by expert panels and health organizations for guidance in managing this condition (e.g. EUROMENE) [89]. Among these, the **UK’s National Institute for Health and Care Excellence (NICE)** released a comprehensive guideline in 2021 that has been highly influential [90]. In the United States, an authoritative report by the **Institute of Medicine** (now National Academy of Medicine) in 2015 provided a framework for understanding and managing ME/CFS, although no official U.S. clinical practice guideline per se has been established [91, 92]. The U.S. Center for Disease Control and Prevention (CDC) provides informational resources emphasizing symptom management and pacing but also notes that no FDA-approved treatments exist for ME/CFS [93].

**NICE Guideline (2021)**: The 2021 NICE guideline on ME/CFS represents a significant shift in recommendations [94]. It acknowledges ME/CFS as a serious, chronic medical condition that can be profoundly disabling, and it calls for individualized, multidisciplinary management. Importantly, the guideline *discourages previously recommended interventions like graded exercise therapy (GET)* – any program based on fixed incremental increases in activity or exercise should *not* be offered, due to the risk of exacerbating symptoms (especially **post-exertional malaise**, a hallmark of ME/CFS). Instead, patients are advised to engage in “energy management” or pacing: balancing activity and rest to avoid overexertion. **Cognitive-behavioural therapy (CBT)**, which earlier guidelines had positioned as a primary treatment, is now recommended only as an *adjunct supportive therapy* – for helping patients cope with the illness, not as a cure or means to dismiss the underlying disease. The NICE guidance emphasizes a collaborative care plan focusing on symptom management (e.g. pain, sleep disturbances, orthostatic intolerance, depression or anxiety if present) and ensuring any interventions are tailored to the individual’s needs and tolerances. Pharmacologic treatments are generally directed at comorbid symptoms – for instance, low-dose antidepressants for co-occurring depression or pain, or sleep medications for insomnia – **but there is no specific drug treatment for the fatigue of ME/CFS itself** [94]. Patient education and validation are stressed: clinicians should acknowledge the reality and severity of the illness, involve patients in shared decision-making, and guard against stigma or psychologization of symptoms. Another noteworthy change is that ME/CFS can now be diagnosed after 3 months of persistent symptoms (rather than 6 months), to facilitate earlier intervention [90]. Overall, the NICE 2021 guidelines mark a more conservative and patient-centered approach, likely reflecting patient advocacy input and evolving evidence.

**Institute of Medicine Report (2015)**: In the absence of APA/WPA guidelines, the IOM’s expert panel report serves as a de facto guide in the U.S [91]. The IOM re-defined ME/CFS with the term “systemic exertion intolerance disease (SEID)” and established updated diagnostic criteria, underscoring core features like post-exertional malaise, unrefreshing sleep, cognitive impairment (“brain fog”), and orthostatic intolerance. The report validated that ME/CFS is a **real**,** biological illness** – not a merely psychosomatic condition – and called for greater clinician education and research investment. For management, the IOM similarly highlighted pacing of activities, symptomatic treatments, and CBT as a supportive therapy. It did *not* endorse any specific pharmacotherapy for fatigue, noting that trials of drugs such as rintatolimod or various stimulants had shown inconsistent results. The IOM’s conclusions align with the current ethos: management should be personalized, holistic, and supportive. The CDC’s recommendations echo these principles [93], emphasizing adequate rest, *activity management (pacing)*, counselling for coping strategies, and treating comorbid issues. Patients may benefit from **psychological support** (e.g. therapy or support groups) to help cope with the impact of chronic fatigue on mental health and daily function (Abrahams et al., 2018). Psychiatrists can play a key role in addressing secondary depression or anxiety, and in working as part of a multidisciplinary team to support the patient’s overall well-being.

In summary, **official guidelines for chronic fatigue (ME/CFS)** stress a cautious, patient-tailored approach: *diagnose early*,* rule out other causes*,* validate the patient’s experience*,* and focus on improving quality of life through pacing and symptom management*. Overly aggressive exercise programs are contraindicated, and while behavioural interventions like CBT or graded activity had been mainstays in older guidance, they are now considered supportive at best (and potentially harmful if misapplied)​ [94]. The shift in ME/CFS guidelines highlights the field’s recognition that chronic fatigue in this context has multifactorial biological underpinnings, requiring a management strategy distinct from the approach to fatigue in other illnesses such as cancer. To synthesize the above, Table 4 presents key points from prominent official guidance on ME/CFS.\.

**Table 4:** Official guidelines and consensus recommendations for chronic fatigue syndrome (ME/CFS). These emphasize individualized, supportive care and caution against aggressive exercise-based therapies

Guideline / Source	Organization (Country)	Year	Key Recommendations for Chronic Fatigue (ME/CFS)
NICE Guideline NG206 on ME/CFS	NICE (UK)	2021	Diagnose after ≥ 3 months of unexplained disabling fatigue; confirm presence of core symptoms (post-exertional malaise, unrefreshing sleep, cognitive difficulties, orthostatic intolerance). Emphasizes personalized energy management (pacing) – balance rest and activity to avoid crashes. Graded Exercise Therapy (GET) is not recommended (no fixed incremental exercise programs) due to risk of harm. Cognitive Behavioural Therapy (CBT) is offered only as an adjunct for coping (not as a curative treatment). A multidisciplinary care plan should address symptom relief (pain, sleep, mental health, etc.), with regular reviews. No specific medication for fatigue; pharmacotherapy directed at comorbid issues (e.g. depression, insomnia, pain) on an individual basis. Educate and support patients and caregivers; acknowledge the biological nature of illness and avoid implying it’s “all in the mind.”
Institute of Medicine (IOM) Report	National Academy of Medicine (USA)	2015	Redefined ME/CFS (also proposed name SEID) and set modern diagnostic criteria. Stressed that ME/CFS is a legitimate medical condition involving profound fatigue and post-exertional symptom exacerbation. Recommended comprehensive evaluation to exclude other causes. Management approach: “Stop–Rest–Pace” – patients should listen to their body’s limits; utilize pacing to prevent post-exertional malaise. No curative treatment; focus on symptom-based interventions: e.g. low-dose antidepressants or cognitive therapy for secondary depression/anxiety, sleep hygiene or medications for insomnia, compression garments or salt/fluid intake for orthostatic intolerance, etc. Advised against vigorous exercise regimens; instead encourage gentle, tolerable activity as able (without causing relapse). Highlighted need for healthcare provider education to improve early recognition and supportive care.
(No specific APA/WPA guideline)	(N/A – rely on above sources)	–	The APA and WPA have not issued standalone guidelines for chronic fatigue syndrome. Psychiatrists refer to general guidelines (like NICE) and emerging research. Standard psychiatric practice is to help manage comorbid psychiatric symptoms (e.g. treating clinical depression or anxiety that often accompany ME/CFS) and to collaborate with other specialists. Emphasis is placed on validating the patient’s experience and avoiding inappropriate psychiatric labeling of ME/CFS itself. Recent guidance encourages viewing chronic fatigue through a biopsychosocial lens without minimizing its physical aspects.

### Comparison of ME/CFS Guidelines Vs. Oncology Guidelines for Fatigue

Chronic fatigue in *psychiatric/primary care contexts* (i.e. ME/CFS) is managed differently from **cancer-related fatigue (CRF)** in oncology, though there are some overlaps. A comparative look at guidelines for each reveals key differences in recommended interventions, reflecting the distinct nature of these conditions.

**Scope and Underlying Etiology**: ME/CFS is a standalone chronic illness of unclear etiology (thought to involve immune, neurological, and endocrinological dysregulation). In contrast, CRF is a *secondary symptom* in patients with cancer or cancer survivors, arising from cancer and its treatments (chemotherapy administration, radiation-induced, surgery, hormonal treatment, etc.)​ [95]. Oncology guidelines for instance those from the National Comprehensive Cancer Network (NCCN), American Society of Clinical Oncology (ASCO), and European Society for Medical Oncology (ESMO) explicitly frame fatigue as a consequence of cancer and treatment-related factors [2, 64]. This difference in context influences management: CRF guidelines assume that any ongoing active disease or treatment side effects may be contributing and thus often include **screening for co-existing medical contributors** as a first step [2]. In ME/CFS, by definition, other medical causes of fatigue have been excluded, and the condition itself is the primary focus [96].

#### Exercise and Activity

One of the starkest differences lies in recommendations around exercise. Oncology guidelines strongly endorse **physical activity** and structured **exercise programs** as first-line management for cancer-related fatigue [23, 35]. Supervised aerobic exercise or resistance training, tailored to the patient’s ability, has consistently been shown to reduce CRF and improve functional outcomes, both during treatment and in survivorship [50]. Therefore, NCCN and ASCO guidelines encourage patients to remain as physically active as possible and often refer to exercise as “the evidence-based intervention with the strongest efficacy” for CRF [97–99]. By contrast, in ME/CFS, **graded exercise therapy (GET)** has *fallen out of favour* due to the phenomenon of **post-exertional malaise**– patients can experience a severe exacerbation of symptoms after even minor exertion [94]. As noted, the latest ME/CFS guidance advises against fixed-increment exercise increases and instead focuses on *pacing*. While **gentle stretching and mobility** are not discouraged in ME/CFS, any activity must be carefully self-regulated by the patient. This is essentially the opposite of the cancer-fatigue approach, where pushing to increase exercise (within safe limits) is encouraged in survivors [64]. Thus, what is therapy in one context (exercise for CRF) can be harmful in the other (ME/CFS) and hence clear diagnostic distinction should be meticulously applied.

#### Psychological Interventions

Both ME/CFS and CRF guidelines incorporate psychological interventions, but with different emphases. In ME/CFS, **CBT** is offered as a supportive measure to help patients cope with the illness’s impact, and to manage stress or unhelpful illness beliefs, but it is explicitly *not* a cure [90]. Historically, CBT plus GET was the standard care for ME/CFS in some guidelines (e.g., NICE 2007), premised on a now-challenged hypothesis that deconditioning and fear of activity perpetuated fatigue. The new paradigm acknowledges that while CBT can improve patients’ mental health and coping skills, it does still not directly “fix” the underlying fatigue pathology​ [100]. In oncology fatigue guidelines, *psychosocial interventions* are also integral – including CBT, mindfulness-based stress reduction, and counselling – particularly when fatigue coexists with mood disorders or sleep disturbance [64, 101]. The evidence indicates that psychosocial interventions yield modest improvements in CRF, often in conjunction with exercise [50]. For cancer survivors, CBT might focus on energy conservation strategies and addressing triggering factors such as stress or anxiety about cancer recurrence. Both sets of guidelines value psychosocial support, but ME/CFS guidelines tend to **warn against overemphasizing psychological explanations**, whereas oncology guidelines freely incorporate psychological techniques as part of multidisciplinary care without concern of implying the fatigue is “all in the head,” since the cancer etiology is understood [5].

#### Pharmacological Treatments

No **drug has regulatory approval for ME/CFS** fatigue, and guidelines do not recommend any specific pharmacotherapy for the fatigue symptom itself [91, 100]. Psychiatrists may trial medications targeting prominent associated symptoms – e.g., low-dose duloxetine or amitriptyline for fibromyalgia-like pain or sleep, or stimulants in severe refractory cases – but evidence for these in ME/CFS is limited and they are not standard guideline recommendations [102]. On the oncology side, **pharmacologic options for CRF** have been explored and appear in guidelines with cautious endorsement for select patients [103]. For example, **psychostimulants** like methylphenidate or modafinil have shown small short-term benefits in some cancer fatigue trials [58, 104]. Accordingly, the ASCO and NCCN guidelines note that stimulants can be considered only for **refractory CRF** or when rapid improvement in fatigue is needed (e.g., to maintain work schedules), but they refrain from routine recommendation due to mixed evidence and potential side effects.

**Corticosteroids** have been used for fatigue in advanced cancer/palliative settings with temporary benefit but are not suitable for long-term use. Other agents like antidepressants have generally not shown significant benefit on fatigue unless there is a concurrent depression.

In summary, **medications play a minor role** in both conditions’ fatigue management, but oncology guidelines are somewhat more open to off-label pharmacological stimulants in carefully selected cases [2, 64, 101] whereas ME/CFS guidelines focus on symptom-by-symptom management with no specific recommendation for stimulants (which can often lead to “crashes” in ME/CFS patients if not very carefully managed) [105].

#### Multidisciplinary Care and Rehabilitation

Both sets of guidelines underscore a multidisciplinary approach. For cancer survivors with CRF, guidelines often recommend a combination of exercise, psychosocial interventions, and management of medical comorbidities – delivered by a team that may include oncologists, rehabilitation specialists (physical therapists, occupational therapists), psychologists, and palliative care or integrative medicine practitioners [106]. There is an emphasis on **survivorship care plans** that include fatigue monitoring and interventions [107]. In ME/CFS, multidisciplinary care might involve primary care physicians, neurologists or infectious disease specialists familiar with the illness, physiotherapists for very gentle mobility work, and mental health professionals for counselling support. Notably, ME/CFS patients often report difficulty finding knowledgeable providers, which is why guidelines call for specialized ME/CFS clinics or teams where possible [94]. **Patient education** is a common theme: both groups of guidelines encourage educating patients about energy conservation, sleep hygiene, and stress management techniques [108]. However, oncology guidelines also focus on **motivational encouragement** to increase activity and engagement in life roles as recovery continues [64], whereas ME/CFS guidelines prioritize **validating limitations** and avoiding pushing patients beyond their limits [90].

#### Outcome Expectations

Another subtle difference is observed in prognosis and expectations. In the cancer setting, fatigue usually improves in the months following after treatment end, and interventions aim to hasten that improvement and restore survivors to their prior level of functioning [101]. CRF is seen as *time-limited* for many (though a subset of survivors has chronic persistent fatigue for years). In ME/CFS, the course can be protracted for many years or a lifetime, and while some patients improve, others do not; thus, the goals are often about improving quality of life and day-to-day function rather than expecting a full “recovery” in the short term.

Psychiatric guidelines for ME/CFS, implicitly, are about *managing a chronic condition*, whereas oncology guidelines are often about *managing a long-term side effect* in a population whose primary disease may be in remission.

##### Management

It is of importance that a significant subset of patients with CRF **also exhibit post-exertional malaise**,** a seminal symptom of ME/CFS** [109]. This points out that there may be a meaningful comorbidity, promoting the use of strategies that aim to alleviate symptomatology from both nosological entities.

In summary, oncology and psychiatric (ME/CFS) guidelines for fatigue both endorse a **multimodal approach** and recognize the importance of psychosocial support, but they diverge on exercise prescriptions and the conceptualization of the fatigue’s origin. Table 5 emphasizes key differences and overlaps of the psychiatric and oncologic approaches.

**Table 5:** Comparison of oncological vs. psychiatric strategies and approaches to aspects of chronic fatigue, cancer-related or ME/CFS

Aspect	ME/CFS (Psychiatric/General Guidelines)	Cancer-Related Fatigue (Oncology Guidelines)
Context of Fatigue	Primary illness (ME/CFS) – chronic fatigue as the central defining symptom of a neuroimmune disorder (not due to ongoing exertion or another disease).	Secondary symptom – fatigue consequent to cancer and its treatment in patients or survivors (often co-occurring with pain, anaemia, etc. as part of survivorship issues).
Diagnostic Criteria	Requires at least 3–6 months of unexplained, disabling fatigue plus hallmark symptoms (post-exertional malaise, unrefreshing sleep, cognitive impairment, orthostatic intolerance). Diagnosis of exclusion – other medical and psychiatric causes must be ruled out (Institute of Medicine, 2015).	No specific timeframe (fatigue can start during cancer therapy or after). Diagnosed clinically by patient-reported fatigue disproportionate to activity. Guidelines advise routine screening (e.g. NCCN recommends asking every patient about fatigue at visits) and evaluation for contributing factors like anaemia, thyroid dysfunction, depression, etc.
Pathophysiological Emphasis	Dysregulated physiology (immune, autonomic, HPA axis). Post-exertional malaise (worsening of symptoms after activity) is a key feature, suggesting an inability to tolerate physical or cognitive stress. Illness often triggered by infection or other stressor; ongoing research into inflammatory and neurological underpinnings.	Multifactorial causes: cancer itself (tumor byproducts, cytokine release), inflammation, effects of treatments (chemo, radiation, surgery), hormonal changes (e.g. induced menopause), muscle deconditioning, sleep disturbances, pain, emotional distress. No single mechanism – CRF is thought to result from a combination of central (cytokine and neuroendocrine) and peripheral (muscle metabolic) factors.
Role of Exercise	Pacing not graded exercise. Patients are cautioned to avoid overexertion due to post-exertional malaise. Activity is tailored to tolerance; some days may require rest. Structured exercise programs (GET) are not recommended as a treatment (NICE, 2021). The focus is on maintaining basic mobility and preventing deconditioning within the patient’s energy envelope.	Exercise is first-line therapy. Guidelines strongly encourage regular aerobic and resistance exercise to combat deconditioning and physiologic fatigue drivers [23]. Even during chemo, patients are advised to stay active if possible. Prescriptions may include walking, cycling or supervised programs, scaled to fitness level (e.g. starting with low intensity and gradually increasing duration). Exercise is safe and effective for most cancer survivors and can significantly improve fatigue
Psychosocial Interventions	CBT or supportive therapy to aid coping, treat coexistent depression/anxiety, and assist with activity management. Emphasis on validation – therapy acknowledges the genuine physical nature of the illness while helping patients adapt (NICE, 2021). Other modalities like mindfulness, support groups, or relaxation techniques can reduce stress and improve quality of life. Family education is important to create a supportive environment.	CBT and psychosocial support are recommended to address maladaptive thoughts (“fear of exercise,” etc.), improve coping skills, and treat comorbid mood issues [101]. Strategies like energy conservation training (learning to prioritize tasks and rest), stress management, and sleep hygiene are commonly included. Mind-body interventions (yoga, tai chi, mindfulness meditation) have evidence for modest benefits in CRF and are often suggested as adjuncts [33]. Involvement of psycho-oncology services or rehabilitation counselors is encouraged for persistent fatigue.
Pharmacological Approach	No approved drug for fatigue. Treat comorbid conditions individually: e.g. use low-dose antidepressants for co-occurring depression or fibromyalgia-like pain, anxiolytics for severe anxiety or sleep aids for insomnia [93]. Some clinicians may trial stimulants (methylphenidate, modafinil) off-label in severe cases, but this is not standard, and evidence is minimal – careful monitoring is needed as patients can overdo activity on stimulants and “crash” afterwards. Overall, meds provide symptom relief (pain, mood, sleep) rather than directly alleviating fatigue.	No universal drug cure for CRF, but certain medications are utilized in select cases. Psychostimulants (methylphenidate, modafinil) have shown modest improvements in energy and are mentioned in guidelines as an option for short-term use in refractory fatigue [13, 58]. If fatigue is related to hormone changes (e.g. in men on androgen deprivation or women in menopause), addressing those (testosterone supplementation or thyroid hormone if indicated) may help. Erythropoietin-stimulating agents can be used if fatigue is due to chemotherapy-induced anaemia (though concerns about safety limit this to certain patients). Importantly, any uncontrolled symptoms exacerbating fatigue (pain, nausea, depression) should be treated – e.g. adequate pain management can secondarily improve fatigue.
Outlook and Monitoring	Management is typically long-term. Some ME/CFS patients improve over time, but many have a relapsing-remitting or chronic course. Goals are to stabilize daily functioning and prevent post-exertional crashes. Regular follow-ups focus on adjusting the pacing plan and addressing new symptoms or comorbidities. Success is measured in improved quality of life, not necessarily elimination of fatigue [91].	Fatigue usually gradually improves after cancer treatment ends, so there is an expectation of recovery in many cases. Interventions aim to accelerate this recovery and reduce fatigue severity in the interim. Oncologists or survivorship clinics monitor fatigue at each visit (e.g. using 0–10 scales). If fatigue persists or worsens, guidelines prompt re-evaluation for recurrence or late effects. Survivors are encouraged to set incremental fitness goals and return to work/activities as able. A successful outcome is a survivor who is able to resume normal or near-normal activities with manageable fatigue levels

As shown above, **psychiatric (ME/CFS) guidelines and oncology guidelines converge on a holistic approach**, but they diverge in important therapeutic directions. In practical terms, a cancer survivor with chronic fatigue will be encouraged by oncology guidance to *gradually increase physical activity*,* engage in rehabilitation exercise programs*,* and possibly try a short course of a stimulant medication*, alongside psychosocial support [50, 64]. In contrast, a patient with ME/CFS will be advised by current guidelines to *pace their activities to avoid crashes*,* possibly limit exercise to only very mild activity that they can tolerate*,* and focus on managing stress and symptoms*, with no specific drug therapy for their fatigue [90, 91, 93]. One challenge in the psychiatric realm is that if a survivor’s fatigue does not fall neatly under a mental health diagnosis, insurance coverage for therapy might be limited. Additionally, forthcoming classifications may formalize CRF in diagnostic terms – for example, the ICD-11 has an optional code for “Chronic fatigue following neoplastic disease” in the symptoms chapter, which might help legitimise the condition across fields.

Both approaches indicate that fatigue is real and deserves attention, and both encourage **multidisciplinary care** – yet the execution of care differs in line with each condition’s pathophysiology and patient experience. Clinicians need to be aware of these differences to avoid applying the wrong paradigm: for example, treating an ME/CFS patient with the oncology model of vigorous exercise could be detrimental, whereas treating a cancer survivor’s fatigue with only rest and pacing could lead to missed opportunities for improvement. Oncologists historically focus on tumor-directed issues and may under-recognize fatigue, whereas mental health professionals are trained to assess functional and mood aspects – so a psychologist or psychiatrist might be more likely to initiate a conversation about fatigue severity and its emotional toll. Psychiatrists approach CRF within the framework of **central fatigue** or **“vitality”** – concepts familiar in treating conditions like chronic fatigue syndrome or depressive fatigue. They may draw on their experience that stimulants can help some patients with fatigue in other contexts (e.g. multiple sclerosis or post-stroke fatigue) [110, 111] and apply that knowledge here. However, given the ASCO 2024 guidance and lack of robust data, the psychiatric community also exercises caution. Any off-label pharmacotherapy is typically seen as an adjunct to, not replacement for, proven non-drug therapies. In summary, while there isn’t a distinct “psychiatric guideline” that contradicts oncology guidelines, the emphasis might differ: psychiatrists focus on treating comorbid mood/sleep disorders and improving coping skills (which indirectly improves fatigue), and they may be *open to* trying medications like stimulants or SNRIs in refractory cases, whereas oncology guidelines increasingly discourage reliance on these medicines due to inconsistent evidence. Ultimately, both oncology and psychiatric experts agree on the primacy of non-pharmacologic interventions and the importance of a personalized, whole-person approach to managing CRF.

### Future Directions and Gaps

Chronic fatigue in cancer survivors remains an area of active research, and many questions about its pathogenesis and optimal management are still unanswered. Here we outline some key future directions and gaps that need to be addressed:

#### Unravelling Biological Mechanisms

Continued research is needed to better understand the **biological underpinnings** of CRF. While inflammation has been implicated, the precise immune mediators and pathways have yet to be fully elucidated. Future studies are exploring links between persistent inflammation and neuroendocrine changes – for example, examining whether pro-inflammatory cytokine surges during treatment lead to long-term alterations in brain neurotransmitter systems or HPA axis function that sustain fatigue. Researchers are now leveraging advanced techniques (e.g. cytokine profiling, genetic and epigenetic analyses) to identify biomarkers that predispose patients to chronic fatigue. An emerging area is the role of the **gut microbiome** and systemic metabolism in fatigue. Some preliminary evidence suggests the gut microbiome composition might influence inflammation and fatigue in cancer patients​ [112], and interventions targeting the microbiome (dietary changes or probiotics) could potentially modulate fatigue – an intriguing hypothesis yet to be tested. Additionally, **genetic studies** are looking at polymorphisms in inflammatory genes or neurotransmitter genes that might make certain patients more susceptible to CRF [113, 114]. If such biomarkers can be identified, it could enable *risk stratification*: predicting who is likely to develop severe fatigue and implementing early interventions for those individuals [9].

#### Clinical Trials of Novel Interventions

On the therapeutic front, there is a clear need for large, well-designed clinical trials to evaluate interventions for CRF, especially pharmacological ones. Many past trials were small or negative, leaving uncertainty around drugs. Ongoing trials are now addressing some of these gaps. For instance, the **ENERGIZE trial** (a phase III RCT of bupropion vs. placebo for CRF) is underway in the United States [71] – its results will provide higher-level evidence on whether bupropion should be part of routine care. The **5-EPIFAT trial** in Iran, as mentioned, is testing multiple medications head-to-head [73]. In addition, combination strategies are being formally tested. One notable study is an NCI-supported phase III trial [115] in patients with metastatic cancer on immunotherapy, which is evaluating **methylphenidate + a physical activity program vs. placebo + physical activity** for reducing fatigue​. This study will inform whether adding a stimulant to exercise yields additive benefit in a controlled setting. Another trial [116] is examining methylphenidate with exercise in prostate cancer survivors with fatigue. Results from such trials will clarify the role of pharmacologic *augmentation* of exercise. Beyond stimulants, researchers are testing anti-inflammatory approaches: small pilot trials are exploring drugs like **omega-3 fatty acids** (with anti-inflammatory properties) [117] or even specific cytokine inhibitors in cancer survivors with high inflammation and fatigue, although these are in early phases [118]. **Corticosteroids** are also being studied in short courses for intermediate stages (not just end-of-life) to see if they can be used intermittently to “rescue” patients from bouts of severe fatigue; careful attention to side effect trade-offs will be key here.

Another exciting area is **mind-body interventions** and their optimization. While therapies like yoga and mindfulness are effective, questions remain about how to best implement them (e.g., in-person vs. digital delivery, optimal session frequency, etc.) [119, 120]. Trials are investigating technology-assisted interventions – for example, smartphone apps to deliver CBT for fatigue or to monitor activity and symptoms in real-time, providing tailored feedback to patients [121]. These digital health approaches could expand access to effective fatigue management for survivors who cannot attend in-person programs [122].

#### Integrating Fatigue Management into Survivorship Care

A practical gap is that even when effective interventions exist, not all survivors receive them. Surveys indicate many oncologists do not routinely counsel patients on managing fatigue, and patients often feel that this issue is overlooked​ [123]. Future efforts should focus on **implementation**: how to ensure every survivor with significant fatigue is identified and offered evidence-based interventions. This could involve survivorship care plans that automatically include fatigue evaluation and referrals (e.g., to physical therapy or psycho-oncology) for those above a certain fatigue severity. Oncology clinics could be beginning to adopt **screen-and-intervene protocols**: for instance, using tablet-based questionnaires in the waiting room to flag high fatigue, then triggering a referral to a fatigue management program. Continued education of providers is needed so that fatigue is recognized as a treatable condition, not just an inevitable consequence one must endure. Involving rehabilitation medicine and physical therapy early in the survivorship phase is another strategy – a “prehab/rehab” model where patients get structured exercise guidance soon after treatment to prevent long-term fatigue [124].

#### Personalized and Targeted Therapies

The heterogeneous nature of CRF suggests that a one-size-fits-all approach may not be optimal. Future research is trending toward **personalized interventions** [87]. This means identifying subtypes of fatigue – for example, some survivors may have predominantly “central” fatigue (brain fog, motivational deficits, perhaps inflammation-linked) while others have “peripheral” fatigue (muscular exhaustion due to deconditioning or peripheral neuropathy, etc.) [125]. Each subtype might respond to different interventions (a stimulant may help the former, whereas a tailored exercise program addresses the latter). Machine learning analyses of symptom patterns and biomarkers could potentially classify patients into fatigue subgroups, which then inform targeted treatment algorithms. Additionally, **pharmacogenetic** studies might reveal if certain patients are more likely to respond to a given drug (for instance, genetic polymorphisms affecting psychostimulant metabolism or dopamine receptors could influence methylphenidate’s efficacy for an individual) [126]. In the future, one could envision a scenario where a survivor’s blood sample and symptom profile are analyzed and it’s determined that, say, they have high IL-6 and low morning cortisol – indicating an inflammatory fatigue subtype – and a trial of an anti-inflammatory or cortisol-regulating intervention is warranted, versus another patient who has no inflammatory markers but significant psychosocial stress, indicating focus on behavioural therapy.

#### Emerging Pharmacologic Targets

Researchers are also seeking entirely new pharmacologic targets for fatigue. One avenue is the **central nervous system pathways** involved in energy and arousal. Agents that promote wakefulness via novel mechanisms (beyond what modafinil does) are being explored in other conditions and could be tested in CRF. For example, drugs in development for narcolepsy (like those acting on histamine or orexin neurotransmitters) might one day be repurposed if they prove safe in cancer survivors [127, 128]. Another target is the **mitochondrial function** – drugs that support mitochondrial energy production or reduce oxidative stress might help fatigue if mitochondrial dysfunction is confirmed as a contributor. Nutraceuticals like Coenzyme Q10 or nicotinamide riboside (a NAD + precursor) are being studied for general fatigue and could be trialed in cancer survivors [16, 129]. **Anti-inflammatory cytokine inhibitors** (such as IL-1 blockers like anakinra or IL-6 blockers like tocilizumab) have shown some success in treating fatigue in chronic inflammatory diseases (e.g., rheumatoid arthritis); small pilot studies in cancer survivors with elevated inflammatory markers might be a logical next step, though careful attention to infection risks would be needed [130]. Indeed, a 2018 meta-review noted that trials of TNF inhibitors for CRF so far have been inconclusive [131], but as immunotherapies and inflammation-related toxicities become more common in oncology, interest in immune modulators for fatigue may rise. For example, persistent fatigue in survivors of checkpoint inhibitor immunotherapy might be related to immune dysregulation.

#### Addressing Special Populations and Long-Term Outcomes

Future research should also address CRF in **special survivor populations**. For instance, older cancer survivors (age ≥ 65) may have fatigue compounded by aging and comorbidities [47, 132] – what interventions work best for them? Early data suggest exercise is still beneficial in older survivors, but more guidance is needed on safe regimens for those with limitations​ [133–135]. Conversely, younger survivors who may be juggling work and family could benefit from flexible, technology-based interventions (like online fatigue management programs) – such programs are under development and need evaluation for efficacy [121]. Additionally, most studies focus on the first 1–5 years after treatment, but what about survivors 10–15 years out who still have fatigue? Longitudinal studies to understand the trajectory of CRF with a long-term focus will help determine how long interventions should continue and whether late-onset fatigue occurs.

#### Enhancing Collaboration Between Oncology and Psychiatry

Another future direction is fostering collaboration between oncologists, psychiatrists, and other specialists in managing CRF. As seen in the guidelines overview, an interdisciplinary approach is crucial [33, 49]. Psychiatrists can contribute expertise in treating coexistent depression or anxiety and in the judicious use of psycho-stimulant medications when appropriate, while oncologists and rehabilitation specialists focus on medical and exercise interventions. Future guidelines will likely be co-developed by oncology and psycho-oncology experts (as was the case with the ASCO–SIO guideline) to ensure all aspects are covered [64]. The role of **palliative care** and **survivorship clinics** in fatigue management is expanding – these settings are ideal for implementing comprehensive fatigue care, and research can evaluate models where survivors attend specialized fatigue clinics vs. usual care.

In summary, future research aims to close the gap between knowing *what* helps CRF and reliably delivering those interventions to the survivors who need them. There is optimism that with ongoing trials and growing awareness, the next decade will bring clearer answers. As Berger et al. noted, increased funding and prioritization of symptom management research (by NCI, ASCO, and others) is now in place [2]​, which should accelerate progress. The goal is to reach a point where **chronic fatigue is preventable or easily manageable** for all cancer survivors – turning it from a common debilitating complaint into a rarer and more readily treated condition.

Encouragingly, the growing recognition of CRF has led to increased funding and research initiatives. Organizations like the U.S. National Cancer Institute (NCI) and ASCO have identified fatigue as a priority in survivorship research agendas. This has resulted in more large-scale trials and inclusion of fatigue endpoints in many studies (for example, cancer drug trials now often include patient-reported fatigue as an outcome, reflecting its importance). In the coming years, we may see **guideline updates** incorporating new evidence – potentially recommending specific medications if strong data emerges or offering refined exercise prescriptions (such as what intensity or type is optimal). The field is also exploring **preventive** approaches: can we reduce the risk of chronic fatigue by intervening *during* cancer treatment? Trials of exercise during chemotherapy (like the ACT trial) [116] suggest patients who stay active during treatment report less fatigue long-term. Additionally, managing acute fatigue aggressively might prevent it from becoming chronic, though this needs formal study.

Several *research gaps* remain open: the impact of nutrition on CRF (beyond correcting deficiencies) is not well understood – e.g., could an anti-inflammatory diet help? The role of cognitive training or neurostimulatory devices (like transcranial magnetic stimulation) in alleviating fatigue is an emerging area. Furthermore, most CRF studies have focused on common cancers (breast, etc.) and predominantly female samples; more data is needed in male survivors and those with other malignancies (like hematologic cancers, where fatigue after bone marrow transplant is very common). Tailoring interventions to diverse cultural and socioeconomic backgrounds will also be important, as perceptions of fatigue and access to resources can vary widely.

In summary, future directions will likely include developing *targeted pharmacologic treatments* (perhaps inflammatory or neuromodulatory agents) for CRF; optimizing *integrated intervention programs* that address multiple facets of fatigue; harnessing *technology* to support patients in self-management; and implementing *system-level changes* so that every survivor with fatigue is identified and offered effective care. As evidence evolves, clinical guidelines will be updated to reflect best practices​. The ultimate goal is to alleviate CRF to the greatest extent possible for all survivors. Achieving this will require closing the gap between research and practice – ensuring that what we already know (e.g., about exercise and CBT efficacy) is put into action widely, while continuing to discover new solutions for those who remain fatigued.

## Conclusion

Chronic fatigue in cancer survivors is a prevalent and challenging problem, but it is one that can be addressed with a multifaceted approach. Epidemiologically, about one in three survivors experiences persistent fatigue that significantly impairs their quality of life​ [136]. The etiology is complex – involving sustained inflammation and immune changes triggered by cancer therapy, neuroendocrine alterations, and psychosocial factors – which means that effective management must target multiple domains​ [137]. No single intervention is a cure-all for CRF. Instead, the evidence and expert consensus point to a combination of strategies as the best practice. First-line interventions are **non-pharmacological**: dedicated exercise programs, psychosocial support (e.g. CBT, mindfulness), and management of comorbid symptoms can together produce meaningful improvements in fatigue and function​ [64]. Pharmacological treatments, such as stimulants or bupropion, may provide additional relief for some patients but generally yield modest benefits and should be used judiciously, if at all​. Major guidelines now emphasize that medications are *adjuncts* at best and not routine therapy for CRF​ [33, 64, 138]. In all cases, individualization is key – the plan should be tailored to the survivor’s specific needs, whether that means focusing on physical rehabilitation, treating underlying depression, or trying an energy-boosting medication in a carefully selected scenario​.

Crucially, survivors and clinicians should communicate openly about fatigue. Simply acknowledging that fatigue is a real and biologically based consequence of cancer (and not a personal weakness) can validate patients’ experiences and open the door to intervention​ [123]. Effective fatigue management can significantly enhance a survivor’s daily functioning, mood, and return to normal life roles. As our review highlights, when providers **leverage a wide array of therapies – from exercise programs to counselling to**,** in select cases**,** medications – significant reductions in fatigue are achievable**, which in turn improves overall well-being and survivorship outcomes​ [139]. There are still gaps in our knowledge, but ongoing research and clinical innovation are rapidly contributing new insights. In the coming years, we anticipate more refined treatments and perhaps even preventative approaches for CRF (for example, prehabilitation exercise during chemotherapy might reduce post-treatment fatigue).

For now, the take-home message is that **chronic fatigue should be proactively managed as a standard part of cancer survivorship care**. By following evidence-based guidelines, adopting a multidisciplinary approach, and staying attuned to emerging therapies, healthcare providers can help cancer survivors reclaim their energy and vitality to the greatest extent possible. Survivors, in turn, should feel empowered to report fatigue and seek help, knowing there are concrete steps that can be taken. In summary, while chronic fatigue in cancer survivors is complex, it is far from untreatable. With comprehensive care and continued research progress, most survivors can expect to achieve improvements in fatigue and enjoy a better quality of life after cancer​ [138]. The ongoing commitment of the oncology and research communities to address CRF provides hope that the “new normal” for future survivors will include not chronic exhaustion, but a full and active life beyond cancer.

## Key References

- Bower JE, Lacchetti C, Alici Y, Barton DL, Bruner D, Canin BE et al. Management of Fatigue in Adult Survivors of Cancer: ASCO-Society for Integrative Oncology Guideline Update. J Clin Oncol. 2024;42(20):2456-87. 10.1200/JCO.24.00541.
  - Seminal guidelines for the management of CRF in oncological settings.
- Yennurajalingam S, Lu Z, Rozman De Moraes A, Tull NN, Kubiak MJ, Geng Y et al. Meta-Analysis of Pharmacological, Nutraceutical and Phytopharmaceutical Interventions for the Treatment of Cancer Related Fatigue. Cancers (Basel). 2022;15(1). 10.3390/cancers15010091.
- Sun X, Chen Y, Cheung WKW, Wu IXY, Xiao F, Chung VCH. Pharmacological interventions for the management of cancer-related fatigue among cancer survivors: Systematic review and meta-analysis. Integrative Cancer Therapies. 2021;20:15347354211038008.
  - The two largest to date meta-analyses of medicinal interventions of CRF, yielding only moderate effect sizes with medium-to-low quality studies, therefore refraining from suggesting specific medications.
- Garcia-Gonzalez D, Romero-Elias M, Alvarez-Bustos A, Rosado-Garcia S, Sanchez-Lopez AJ, Cantos B et al. Cancer-Related Fatigue and Circulating Biomarkers in Breast Cancer Survivors. Biol Res Nurs. 2024;26(2):270-8. 10.1177/10998004231215777.
  - An important overview of Immune, hormonal and behavioural factors of CRF.
- Toogood PL, Clauw DJ, Phadke S, Hoffman D. Myalgic encephalomyelitis/chronic fatigue syndrome (ME/CFS): Where will the drugs come from? Pharmacol Res. 2021;165:105465. 10.1016/j.phrs.2021.105465.
  - Mechanistic basis of ME/CFS and potential shared basis with identified immune and hormonal facets of CRF, especially in immune dysregulation.
- Twomey R, Yeung ST, Wrightson JG, Millet GY, Culos-Reed SN. Post-exertional Malaise in People With Chronic Cancer-Related Fatigue. Journal of Pain and Symptom Management. 2020;60(2):407 − 16. 10.1016/j.jpainsymman.2020.02.012.
  - An influential and paradigm altering study showing that the two conditions might overlap and clinicians should be able to identify this co-incidence as it might be course-altering.

## Data Availability

No datasets were generated or analysed during the current study.
